# The roughening kinetics of hydrogenated graphene

**DOI:** 10.1038/s41598-018-27026-8

**Published:** 2018-06-08

**Authors:** S. Son, J. Figueira Nunes, Y. Shin, J-H. Lee, C. Casiraghi

**Affiliations:** 1 School of Physics and Astronomy, University of Manchester, Manchester, M13 9PL UK; 2 National Graphene Institute, University of Manchester, Manchester, M13 9PL UK; 3 School of Chemistry, University of Manchester, Manchester, M13 9PL UK; 4 Department of Energy Systems Research and Department of Materials Science and Engineering, Ajou University, Suwon, 16499 Republic of Korea

## Abstract

The roughness is a common property of all growing surfaces – however, the way the roughness of a growing surface changes with time and space is uniquely related to the underlying growth process, i.e. to how the atoms stick to the surface during the first stage of nucleation. This concept allows getting insights on the nucleation process of a growing surface by measuring two scaling exponents, α and β, known as roughness and growth exponents, respectively. In this work, we studied hydrogenation of graphene using the roughening kinetics. The coverage of graphene will depend on how the H ions stick on the surface, giving rise to a unique roughness evolution in time and space. We measured a roughness exponent of ~0.5 (derived from a Fourier index of ~3), and a growth exponent of ~0.3. The values of the growth and roughness exponents are close to those reported for clustered carbon, suggesting a roughening mechanism by clustering, in good agreement with the theory. We also compared our coverage data with a different model, used to describe the dynamics of graphene coverage, during chemical vapour deposition. Our data are in agreement with a nucleation-dominated growth, further confirming that hydrogenation is happening by clustering.

## Introduction

**Figure 1:**
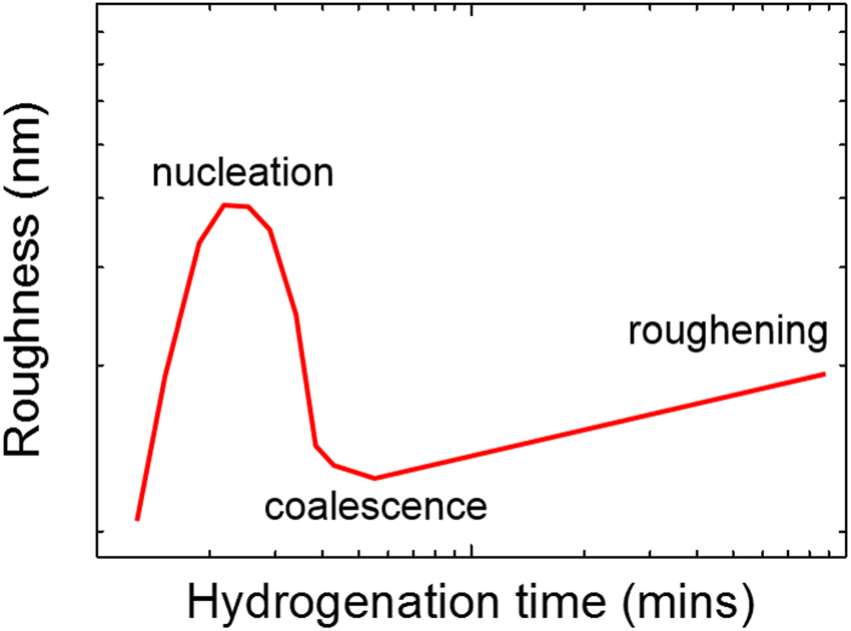
Schematic showing the evolution of the roughness as a function of time for thin films nucleation.

Fractal geometry has been used to describe phenomena such as crystal growth, biological growth and rock fracture, which are too disordered to be studied with other mathematical tools, but still hold a form of order in a scale-invariance^1,2^. This formalism has become a standard tool to study growing surfaces and interfaces^3^: films deposited under non-equilibrium conditions are expected to have surfaces that show different scale-invariant properties along different directions, as the growth process is determined by the competition between fluctuation and relaxation processes, allowing the surface roughness to be characterised by scale-independent parameters. In the framework of the fractal analysis concepts like roughness are replaced by exponents. These exponents do not refer to the roughness itself, but to the way in which the roughness changes when the observation scale itself changes. The knowledge of these scaling exponents allows one to get insights on the growth mechanism of thin films of metals and semiconductors^4–10^ because the evolution of the surface topography at the nucleation stage only depends on how and where a new particle is allowed to come to rest and stick to the existing deposit^2^. Thin films, for example, are expected to nucleate by islands, which then grow in size till reaching coalescence, after which the film roughness saturates, giving rise to the roughness time-evolution shown in Fig. 1.

Graphene is the most famous 2-dimensional crystal with outstanding physical properties^11^ that allows this novel material to be used in a wide range of applications^12^. One of the most attractive advantages of graphene is the possibility to finely tune its properties by covalent functionalization through chemical reactions or plasma exposure^13–15^. In particular, hydrogenation by plasma exposure has been one of the first approaches used to functionalize graphene^14^. Theoretically, the hydrogenation of pristine graphene is not possible, as the elastic energy gain associated with moving atoms out of the graphene’s plane is too high^14^. The buckling deformation caused by the orbital geometry change from coplanar to tetrahedral involves a substantial energy gain to the system, making the re-hybridisation process energetically unfavourable. The C–H bond formation leads to a change in the local atomic structure, producing a strained and buckled surface around the C–H bond, lowering the activation energy to hydrogenation. This local area is considered the “nucleation point” for the hydrogenation to start: once a C–H bond is formed, this acts as a defect by seeding the occurrence of further hydrogenation events around the initial C–H bond. Therefore, hydrogenation is expected to happen by clustering^16^. However, this model has never been fully confirmed experimentally, and the exact hydrogenation mechanism is still under discussion^17–21^.

Although the hydrogenation process is not comparable to the growth of a thin film, the mechanism of hydrogenation by clustering can be described within the thin films nucleation models at the very early stages. If hydrogenation happens by clustering, then the roughness time-evolution is expected to follow the behaviour shown in Fig. 1. In this work we study the hydrogenation process of graphene using the roughening theory. Because graphene is one-atom thick, its surface topography can be strongly affected by the substrate, leading to artefacts in the study of the roughness evolution. Therefore, two substrates have been investigated: silicon and hexagonal-Boron Nitride (h-BN). The ultra-smoothness of h-BN allowed us to analyse in details how the roughness of graphene changes as a function of hydrogenation coverage (in time and space), enabling the measurements of the scaling exponents of hydrogenated graphene. We found a growth exponent of ~0.3 and a Fourier index of ~3, giving rise to a roughness exponent of ~0.5. Based on the Fourier index, the hydrogenation process can be described by the equilibrium between the chemical potentials of two clusters, which seems to be reasonable as hydrogenation is driven by a chemical reaction. The growth and roughness exponents measured are close to those experimentally reported for clustered carbon, confirming a clustering-driven growth process. We finally compared our data with the model used to describe the growth of graphene by chemical vapour deposition (CVD), which is another process characterized by islands growth – we found that the data can be well described by the island nucleation model.

## Scaling Theory

One of the most used parameters, describing the properties of a surface, is the root mean square (RMS) roughness, *R*_q_, defined as^22,23^:

1 $${R}_{{\rm{q}}}=\frac{\sqrt{{{\sum }_{i=1}^{N}[{h}_{i}(x,y)-\overline{h}]}^{2}}}{N}$$

where *h*_*i*_(*x, y*) is the profile function defined at the point of coordinates (*x*, *y*) on the surface, *N* is the number of points, and $\overline{h}$ is the average height value. Note that the roughness contains information about the height distribution, but it does not provide any information on the distance between the features on the surface. The scaling method, based on fractal theory, involves measuring the surface’s roughness at various times and at various radial lengths. This allows replacing the roughness with scale independent parameters, which show how the roughness changes as a function of space and time. This replacement eliminates instrumental dependences such as sampling interval, resolution and so on; indeed, the scaling exponents are determined only by the processes happening during nucleation of the film^24^.

In the framework of the scaling theory, the *R*_q_ of a sample of size *L* × *L* is expected to depend on the measurements window size, *l* × *l* (assuming *l* << *L*) as^2^:

2 $${R}_{{\rm{q}}}(l) \sim {l}^{\alpha },\,for\,l < {l}_{sat}$$

where *l*_sat_ is the window size at which the surface roughness does not change anymore because all surface features are correlated. The roughness exponent, α, is a number between 0 and 1^2^, and can be calculated graphically from the slope of the logarithmic plot of the RMS roughness as a function of window size.

The time-dependent dynamics of the roughening process is described by^2^:

3 $${R}_{{\rm{q}}}(L,\,t) \sim {t}^{\beta },\,{\rm{for}}\,t < {t}_{{\rm{sat}}}$$

where *t*_sat_ is the time required to reach full surface coverage, hence no roughness variation is expected after this time. Based on Eq. 3, the RMS roughness is expected to increase gradually as t increases, until it reaches saturation. The growth exponent, *β*, can be calculated graphically from the slope of the logarithmic plot of RMS roughness as a function of time for very small times (i.e. at the nucleation).  

The scaling exponents *α* and *β* allow the definition of universal growing models. Four kinetic growth models have been theoretically studied for thin film growth, which depends on how and where particles approach, rest and stick to the surface and (or) to existing particle deposits, and how smoothing and fluctuation effects compete with each other in a particular surface^1^. Experimentally, the scaling parameters have been investigated in the case of few carbon-based materials, such as diamond-like carbons^4,6^ and clustered carbon^25^.

### Frequency analysis

The surface properties can be analysed in the frequency space, e.g. by measuring the power spectra density, *PSD*(*w*), of the surface by Fourier transform, $PSD(w)={\mathscr{F}}(C(r,\,t))$, where *C*(*r*, *t*) is the autocorrelation function, given by an average value of the product of two height measurements at a distance r apart and at fixed time. Herring^26^ described four distinct surface transport mechanisms that reduce surface roughness by using the frequency analysis. These surface kinetic models come from an analysis of the time and amount of material needed to produce a geometrically similar change in two different clusters on the surface. The word “cluster” in Herring’s work refers to any particle or to a number of particles, which have started to grow together^26^ and we used the same definition in our work. Every growth mechanism leaves distinct fingerprints on the topography. We consider two spherical clusters of radius R_1_ and R_2_, respectively, where *R*_2_ = *λR*_1_ and *λ* is a scaling factor. For each growth mechanism there will be a relation between the time *δt*_1_, required to produce a certain change in the cluster 1, and the time *δt*_2_, required to produce a similar change in the cluster 2 of the form: *δt*_2_ = *λ*^*i*^*δt*_1_, where i is an integer, called Fourier index. The dominant growth model depends on the Fourier index value. Four cases have been identified: *i* = 1 is associated to viscous flux of an amorphous material, i.e. it has been assumed that temperature is high enough so that the atoms are reasonably free to rearrange themselves. In the case *i* = 2, there is evaporation-condensation, i.e. the vapour pressure of the two clusters are different and the amount of material evaporated from a cluster and condensed to the other will be different from the amount passing in the reverse direction. When *i* = 3, there is bulk diffusion, where the equilibrium between the chemical potentials of the two clusters are considered. Finally, *i* = 4 is associated to surface diffusion, where the rate of migration over a certain type of surface is proportional to the gradient of the chemical potential.

In order to apply this analysis model, the surface profiles are Fourier analysed and the coefficients for the individual profiles are averaged. If a log-log plot of the integrated power spectrum is a straight line, then the modulus of the slope gives *i*. Note that a correlation between the frequency analysis and the scaling approach is expected, as: *i* = 2(*α* + 1)^27^.

### CVD Growth model

The following kinetic model has been proposed to explain the possible mechanisms of graphene growth as a function of the nucleation density^28^. This model assumes that graphene growth takes place on a hexagonal grid of active sites for graphene formation that represents the active sites for graphene formation on a flat surface^28^. The binding of carbon atoms at the active sites occurs from reactive carbon intermediates C_*x*_H_*y*_ ($x\ge 1$, $y\ge 0$) which are caused by the decomposition of CH_4_ molecules on the Cu surface^29–31^. The variation of the graphene coverage *θ* over time is given by^28^:

4 $$d\theta /dt=sJ\theta [1-\theta (t)]$$

where *J* is the impingement rate at a single site and *s* is the sticking coefficient of reactive carbon species (i.e. the probability of binding at the grid sites). Experimentally, the variation in the graphene growth rate observed shows that s is not constant^32^. For this reason, two sticking coefficients need to be introduced: the first sticking coefficient, *s*_0_, describes the mechanism for capturing the active species at random vacant sites, and it is proportional to the fraction of empty sites given by *s*_0_(1 − *θ*(*t*))^28^. The second sticking coefficient, s_1_, describes the physisorption of active species on top of an already formed graphene island, and it is described by a coverage-dependent sticking coefficient as *s*_1_*θ*(*t*)[1 − *θ*(*t*)]^28^. By introducing these coefficients into Eq. (4), the graphene coverage variation with time is now given by:

5 $$d\theta /dt=J\{{s}_{0}[1-\theta (t)]+{s}_{1}\theta (t)[1-\theta (t)]\}$$

Equation 5 can be re-arranged as^28^:

6 $$d\theta /dt=[1-\theta (t)][{\theta }_{0}+\theta (t)]/[\tau (1+{\theta }_{0})]$$

where *θ*_0_ = *S*_0_/*S*_1_ defines the ratio of the sticking coefficients and describes the scaled binding time at a single adsorption site that depends on both sticking coefficients and the impingement rate, and *τ* = 1/*J*(*S*_0_ + *S*_1_) describes the scaled binding time at a single adsorption site that depends on both sticking coefficients and the impingement rate that is proportional to the concentration of active carbon species determined by the CH_4_ flow rate^28^. The solution of Eq. 6 is^28^:

7 $$\theta (t)=1-(1+{\theta }_{0})/(1+\exp [(t-{t}_{0})/\tau ])$$

where *t*_0_ an integration constant given by *θ*(0) = 0. The solution corresponds to a family of sigmoidal S-shaped growth curves that describe the different growth regimes as a function of the flux of growth species given by *τ* and *θ*_0_. Therefore, the dominant growth mechanism can be inferred from the behaviour of the growth curves that is decided by the ratio of the sticking coefficients *s*_0_/*s*_1_. The two limiting cases of the solution of Eq. 7 occur for *θ*_0_ ≫ 1, corresponding to nucleation-dominated growth, and for *θ*_0_ ≪ 1, corresponding to island growth- dominated regime. In particular, for *θ*_0_ ≫ 1 the solution of Eq. 7 can be approximated to *θ*(*t*) = 1 − exp(−*t*/*τ*), resulting in a highly asymmetric curve. As *θ*_0_ decreases, the growth curve becomes more symmetric, showing an inflection point at *θ*_0_ = 1, and finally gets a fully symmetric sigmoidal profile for *θ*_0_ ≪ 1^28^. This method can be extended to hydrogenation of graphene, by measuring the H coverage as function of the time and looking at the shape of the growth curve.

## Results and Discussion

**Figure 2:**
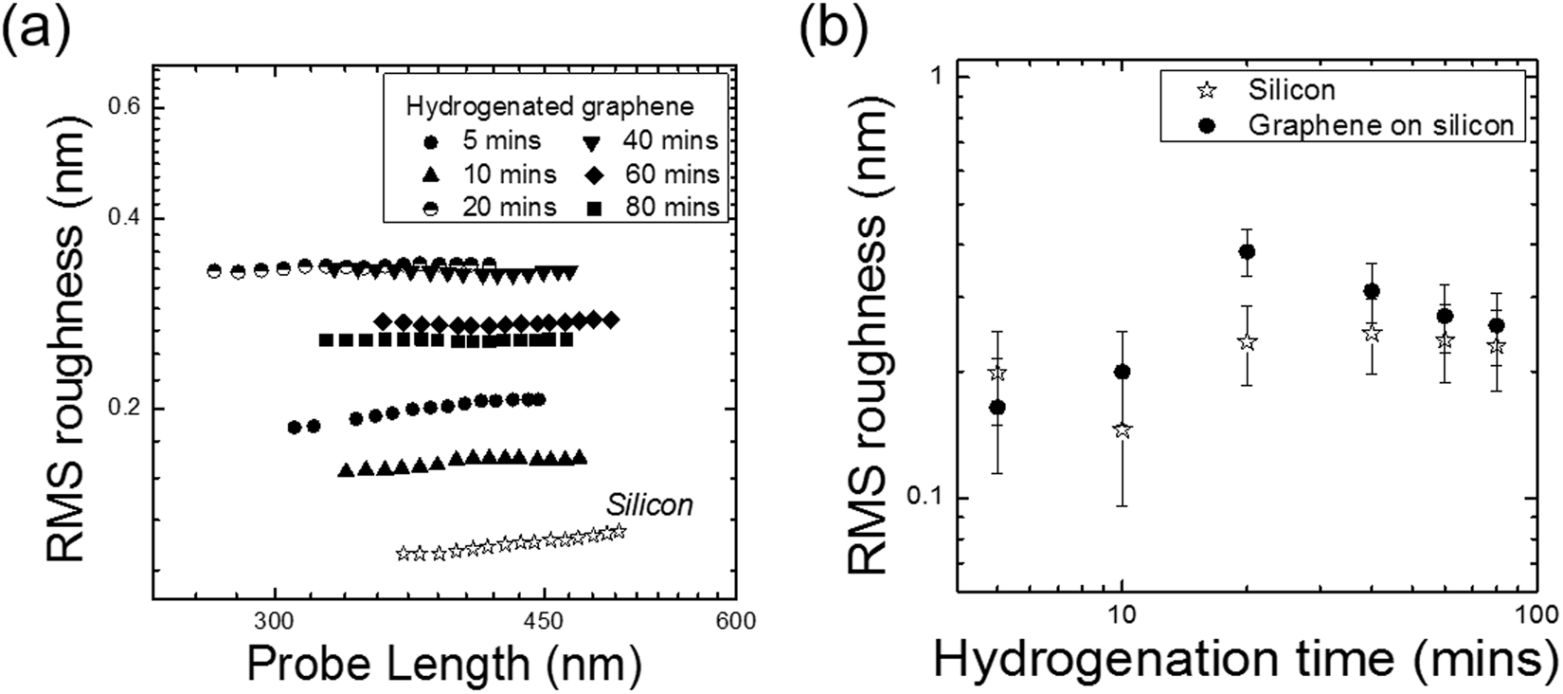
RMS roughness as a function of: (**a**) the probe length for hydrogenated graphene and (**b**) the hydrogenation time for graphene and silicon.

Figure 2(a) shows the RMS roughness of hydrogenated graphene and silicon as a function of the probe length (see Eq. 2). It is clearly seen that the RMS roughness remains constant with respect to the scan-probe length, regardless of the hydrogenation coverage. The same behaviour is observed for silicon. This means that the roughness exponent, α, is zero or that the correlation on the surface can only be observed for probe lengths smaller than 200 nm. One can also see that the RMS roughness slightly changes with the hydrogenation time, from ~0.1 to 0.3 nm, once graphene starts to get hydrogenated, Fig. 2(b); this is expected, as the change from sp^2^ to sp^3^ hybridisation associated with the hydrogenation of graphene involves a planar to tetrahedral geometry rearrangement, with the hydrogen atoms sticking outside the graphene scaffold^14^. However, for increasing times, the RMS roughness oscillates with values all within the experimental error, so it is not possible to clearly see any change in roughness. Figure 2(b) also shows the RMS roughness measured on the exposed silicon substrate, where the sample was transferred. Its RMS roughness remains constant with hydrogenation time: this is expected as the hydrogen plasma can only remove contaminants from silicon’s surface, hence not causing any significant variations in its roughness. More importantly, a part from the point corresponding to 20 minutes hydrogenation, there is no appreciable difference between the RMS of silicon and hydrogenated graphene. These results indicate that the silicon is likely to dominate graphene’s roughness profile, so the scaling exponents cannot be reliably measured. Therefore, a smoother substrate needs to be selected to apply the roughness kinetics. Thus, we replaced silicon with h-BN.

**Figure 3:**
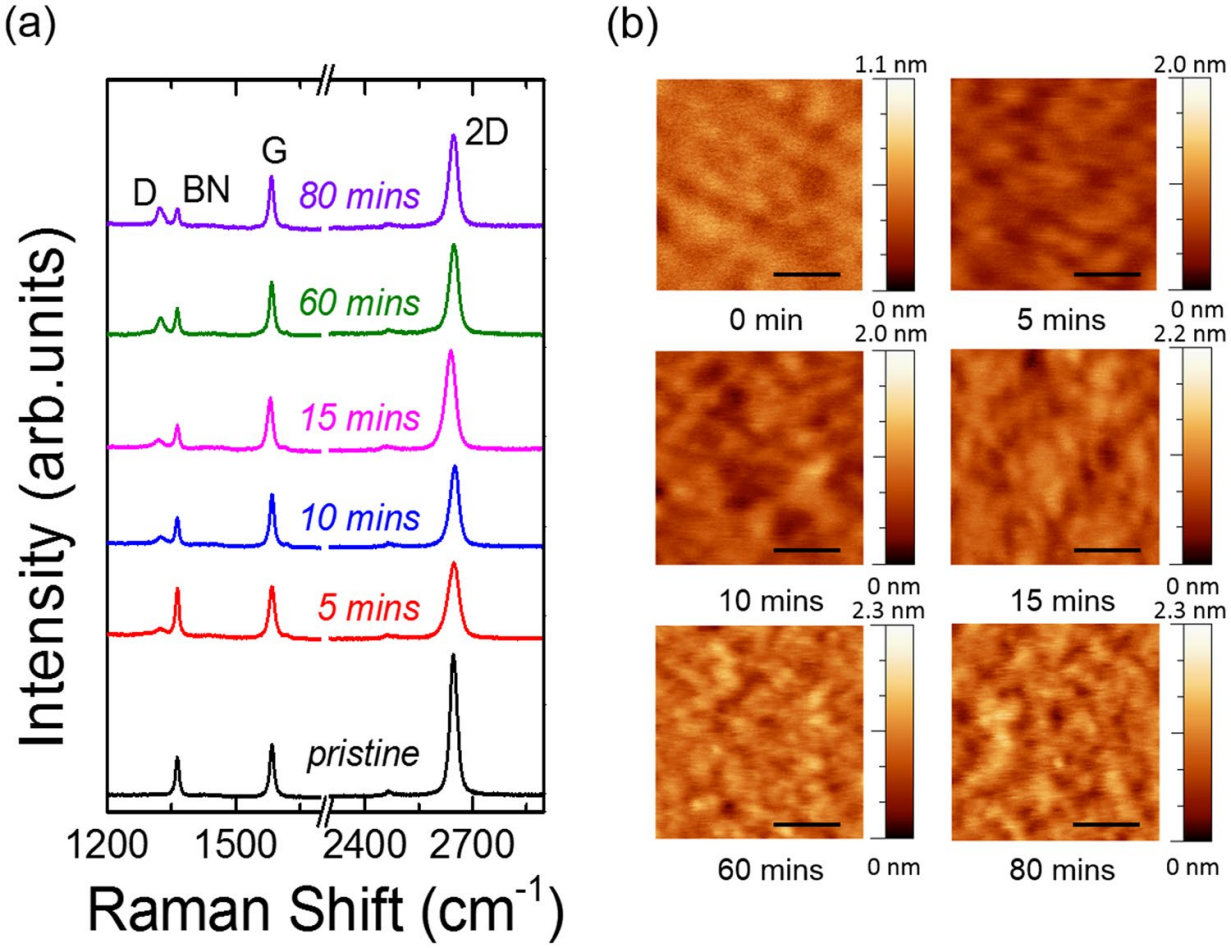
(**a**) Evolution of the Raman spectrum of hydrogenated graphene under different plasma treatment times; (**b**) AFM images of the samples measured by Raman spectroscopy. Scale bar is 300 nm.

Figure 3(a) shows the evolution of the Raman spectrum of hydrogenated graphene on h-BN, obtained under different plasma treatment times. The intensity of the D peak increases with the exposure time, i.e. the intensity ratio between the D and G peaks [*I*(D)/*I*(G)] increases with increasing amount of defects, generated by the longer exposure to the plasma, as observed when low-defect concentrations are introduced in graphene^33–35^. Figure 3(b) shows representative AFM images of hydrogenated graphene obtained under different plasma treatment times.

**Figure 4:**
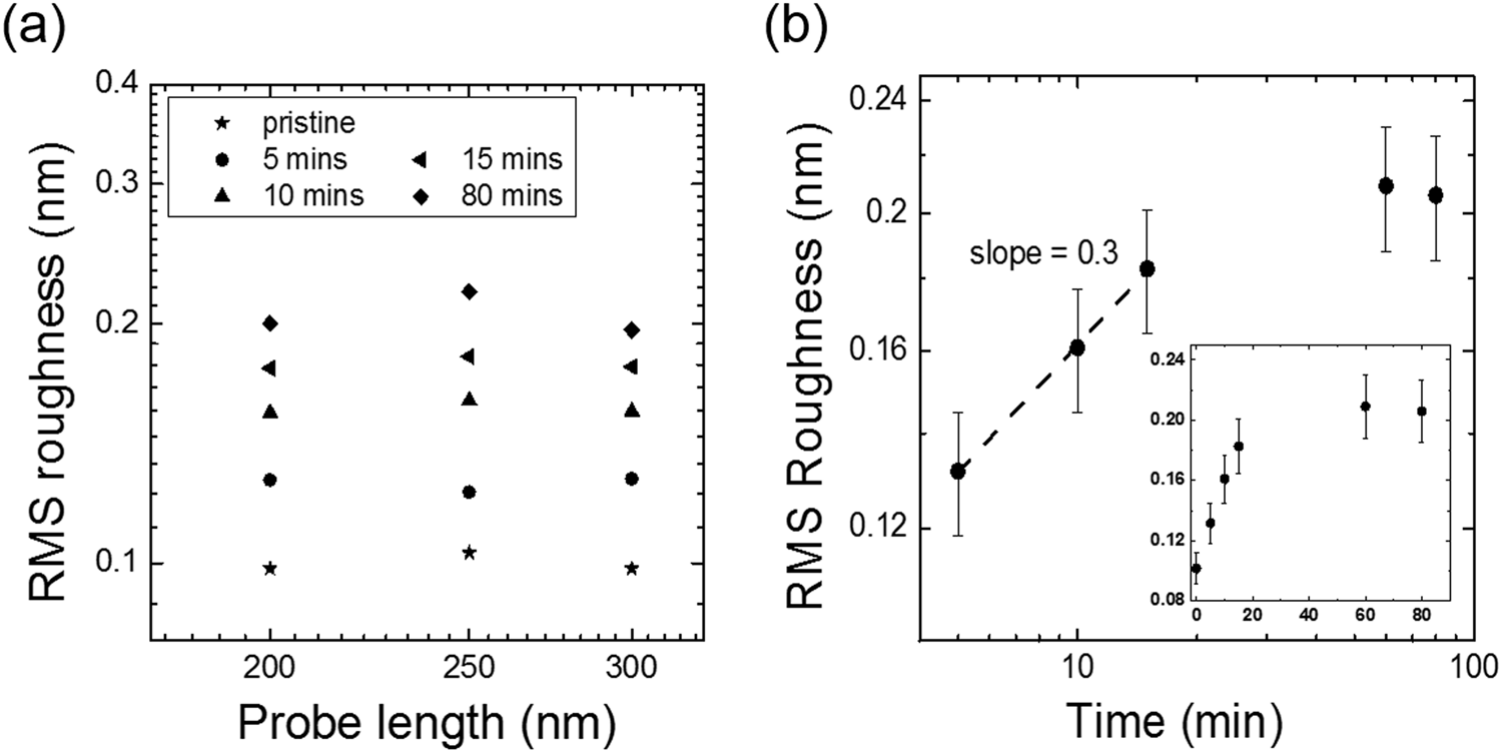
(**a**) A log-log plot of RMS roughness as a function of probe length and (**b**) hydrogenation time for hydrogenated graphene deposited on h-BN. The dotted line is a linear fit between 5 and 15 minutes.

Figure 4(a) shows a log-log plot of the RMS roughness of hydrogenated graphene as a function of scan probe length. As observed for silicon (Fig. 2(a)), we cannot observe any change in roughness and results were not reproducible below 200 nm probe lengths in the case of h-BN.

Figure 4(b) shows a log-log plot of the RMS roughness of graphene as a function of hydrogenation time. The inset in Fig. 4(b) shows the same data on a linear scale to include the RMS roughness of pristine graphene. The RMS roughness of hydrogenated graphene increases in the first 15 minutes, but at longer times (>40 minutes) the RMS roughness seems to reach saturation, in agreement with Eq. 3. A linear regression function was applied to determine the slope of the plot at the initial stage of nucleation for time below 40 minutes. The slope of the plot was found to be ~0.3, which corresponds to the growth exponent *β*. If we compare Fig. 4(b) with Fig. 1, the increase in roughness could be associated to a clustering process; however, no coalescence is observed, i.e. the roughness does not reach a maximum, as shown in Fig. 1. This is in agreement with the clustering model, which does not allow full coverage for one-side hydrogenation (i.e. for supported graphene)^14^.

**Figure 5:**
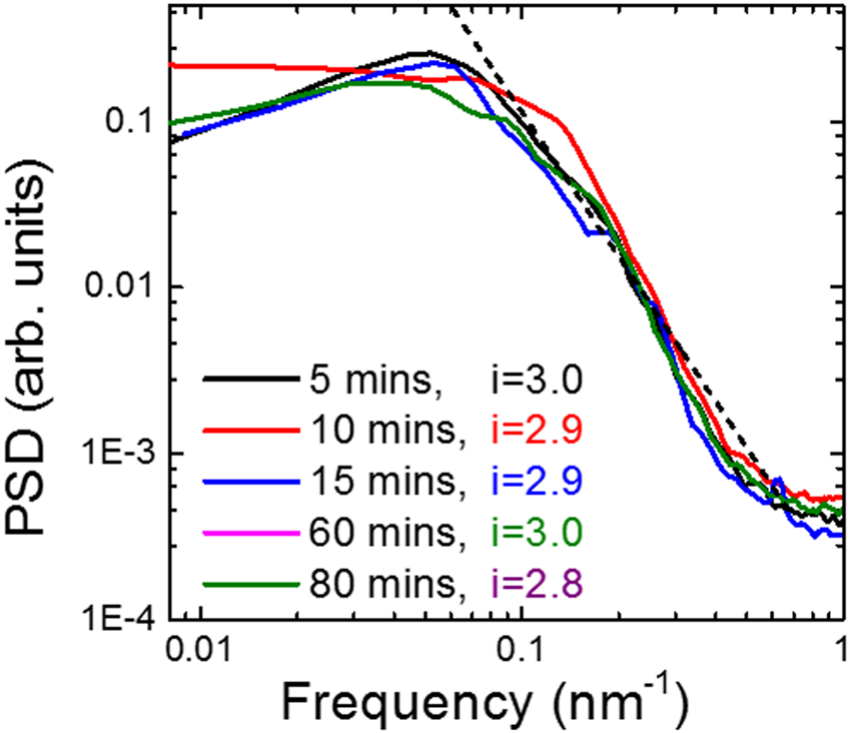
A log-log plot of power spectra density (PSD) of hydrogenated graphene obtained at different hydrogenation times. The slope (dotted line) of the PSD at large frequency gives a Fourier index of ~3.

As the log-log plot of the RMS roughness as a function of the probe length (Fig. 4(a)) could not provide any relevant result, regardless of selected substrates, we then applied the frequency analysis (see the Introduction). Figure 5 shows the PSD of hydrogenated graphene obtained under 5 different plasma exposure time conditions. The Fourier index *i* has been calculated by linear fit of the PSD at large frequency. We found that the value of *i* is between 2.8 and 3.0, giving an average Fourier index of 2.9. The Fourier index of ~3 indicates that the initial stages of hydrogenation are driven by the chemical potential equilibrium, which seems to be reasonable as the hydrogenation process involves a chemical reaction. The Fourier index of ~3 gives $\alpha  \sim 0.5$. The closest exponents *α* and *β* to those we have measured have been reported for clustered carbon^25^, suggesting a clustering-driven mechanism for hydrogenated graphene.

We finally applied the CVD coverage model (see the Introduction). There are two prominent peaks in the Raman spectrum of graphene, known as the G peak and the 2D peak, at ~1580 cm^−1^ and ~2700 cm^−1^, respectively^36^. Raman spectroscopy is strongly sensitive to defects in graphene, as they activate characteristic modes, called D and D′ peaks, at ~1340 cm^−1^ and ~1620 cm^−1^, respectively^33,34^. The Raman spectrum of defective graphene can be described with a phenomenological three-stage amorphization trajectory^7,33,34^. In stage 1, starting from pristine graphene, the Raman spectrum evolves as follows: the D peak appears and *I*(D)/*I*(G) increases; the D′ appears; all the peaks broaden and G and D′ begin to overlap. In this stage, *I*(D)/*I*(G) can be used to estimate the amount of defects^33,35^, while the intensity ratio between D and D′, *I*(D)/*I*(D′), can be used to distinguish between different type of defects^37^. At the end of Stage 1, the G and D′ peaks are no more distinguishable, and *I*(D)/*I*(G) starts decreasing. As the number of defects keeps increasing, the Raman spectrum enters Stage 2, showing a marked decrease in the G peak position and increase broadening of the peaks; *I*(D)/*I*(G) sharply decreases towards zero and second-order peaks are no longer well defined. Stage 3 describes amorphous materials with increasing sp^3^ content^38^. For supported graphene, where only one side of the crystal is available for functionalization, hydrogenation is obtained in stage 1, therefore *I*(D)/*I*(G) increases with increasing amount of defects (i.e. H coverage); this allows us to use *I*(D)/*I*(G) to measure the hydrogenation coverage. Ref.^33^ proposed a relation between defect concentration (n) and *I*(D)/*I*(G) as:

8 $${L}_{{\rm{D}}}^{2}({{\rm{nm}}}^{{\rm{2}}})=(1.8\pm 0.5)\times {10}^{-9}\times {\lambda }_{{\rm{L}}}^{4}\times {[I({\rm{D}})/I({\rm{G}})]}^{-1}$$

and *n*_D_(cm^−2^) = 10^14^/(*πL*_D_), where *L*_D_ is separation between defects, *λ*_L_ is the laser wavelength and *n*_D_ is the number of defects/cm^2^. However, this relation was found for ion-bombarded graphene samples^33^. Assuming that hydrogenation is happening by clustering, then this equation needs to be modified, as shown in ref.^33^, by multiplying *n*_D_ by a correction factor, which takes into account of the clusters formation. By calculating the total number of carbon sites available for hydrogenation as a function of the cluster size (assumed circular in shape and smaller than the defects distance of ~5 nm, which sets the crossing between Stage 1 and 2, a correction factor of ~175 is found^18^, in good agreement with previous works^39,40^.

**Figure 6:**
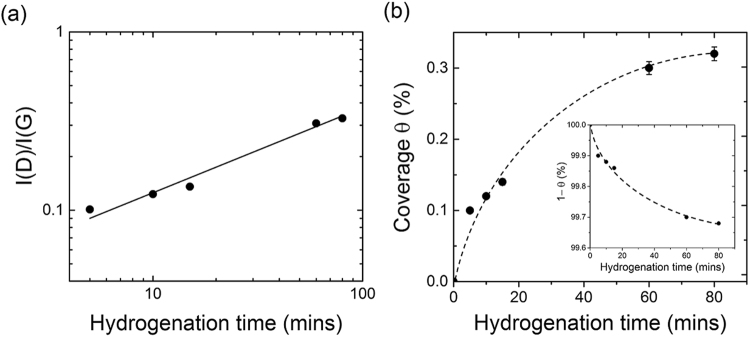
(**a**) A log-log plot of *I*(D)/*I*(G) as a function of the hydrogenation time. The full line is a linear fit of the points obtained between 5 and 15 minutes, giving a slope of 0.48. (**b**) Coverage as a function of hydrogenation time. The dotted line is a guide for the eyes. The inset shows that the experimental data well fit the equation: *θ*(*t*) = 1 − exp(−*t*/τ), which is expected for nucleation-dominated growth.

First, we convert *I*(D)/*I*(G) into coverage, using Eq. 8 and the correction factor. After 5 minutes of hydrogenation, we found that defects are spaced by 54 ± 6 nm; however, after 80 minutes this distance decreased to 30 ± 4 nm, so Stage II is never reached (the distance between defects must be below 3~5 nm^33^). A linear relationship between *I*(D)/*I*(G) and the hydrogenation time was observed in Fig. 6(a). The inset in Fig. 6(b) shows the coverage, extracted from *I*(D)/*I*(G), as a function of the hydrogenation time: this results in a highly asymmetric curve with no inflection point, corresponding to nucleation-dominated growth. Under this regime, the time-dependence coverage should be approximated to: *θ*(*t*) = 1 − exp(−*t*/*τ*). This is confirmed in Fig. 6(b), where the data have been well fitted by the exponential decay.

In conclusion, we applied the roughening theory of thin film to hydrogenated graphenes, in order to get insights on the process driving hydrogenation. A clustering process has been theoretically predicted - this should reflect in a characteristic evolution of the surface properties of graphene for increasing hydrogenation time, as it has been observed for the nucleation of thin films. By looking at how the roughness changes in space and time, we measured a roughness exponent of ~0.5 (derived from the Fourier index of ~3), and a growth exponent of ~0.3. The Fourier index of 3 is associated to a roughening model driven by the chemical potential equilibrium between clusters on the surface. The values of the growth and roughness exponents are very close to those reported for clustered carbon, suggesting a roughening mechanism by clustering. We also compared our data to another model, used to describe the dynamics of the CVD graphene coverage as a function of different experimental parameters. Our data are in agreement with the nucleation-dominated growth, further confirming that hydrogenation is occurring by clustering, without reaching full coverage (for one side hydrogenation).

## Method

The graphene crystals (a few hundreds of microns in size) and thin flakes of h-BN were produced by mechanical exfoliation on a standard silicon substrate with 290 nm SiO_2_^41^. The silicon was cleaned by ultrasonic bath in acetone. The graphene flakes were transferred on silicon and h-BN by using a dry-peel transfer^42^.

The samples were exposed to hydrogen plasma made by using a modified Edwards E306A coating system chamber. A hydrogen-argon mixture (10% H_2_) at a pressure of ~0.1 mbar was employed and a dc plasma ignited between two aluminium electrodes. The samples were placed about 30 cm away from the discharge zone in order to minimise any possible damage due to energetic ions. Each graphene sample was hydrogenated separately at a central position in the sample holder. Thus, all flakes were hydrogenated at the same distance from the hydrogen plasma. Hydrogenation was performed in timescales ranging from 5 to 80 minutes. Note that those experimental conditions were selected in order to have slow hydrogenation to allow us to fully investigate the very first stages of hydrogenation, where the scaling theory can be applied.

Atomic Force Microscopy (AFM) was performed using a Veeco Dimension V in order to investigate the surface properties of the sample. Tapping mode scan over 512 lines was used to collect height information of ranging from ~500 × 500 nm^2^ to ~250 × 250 nm^2^. The Nanoscope Analysis roughness calculator was used to determine the RMS roughness of various areas of graphene. The RMS of silicon and h-BN is ~0.12 nm and ~0.07 nm (Figure S1 in the Supplementary Information). The RMS of pristine graphene on silicon and h-BN (Figure S1 in the Supplementary Information) is 0.17 nm and 0.1 nm, respectively, measured on an area of 500 × 500 nm^2^.

A Renishaw Raman spectrometer, equipped with an excitation lines of 514.5 and 633 nm, was used to identify graphene^36^ and to determine hydrogenation conditions^14^. In all cases, a 100× objective giving a laser spot size of ~400 nm was used. The incident power was maintained below 1 mW during the measurement to avoid any possible damage and heating of samples, which may lead to hydrogen removal.

## Electronic supplementary material

Supporting information
