# Supplementary material for: The roughening kinetics of hydrogenated graphene
Source: Sci Rep. 2018 Jun 8;8:8771. doi: 10.1038/s41598-018-27026-8 (PMC5993728; doi:10.1038/s41598-018-27026-8)
Supplement: Supplementary file 1 — Supporting information [file 41598_2018_27026_MOESM1_ESM.docx]

**Supplementary information for**

**The roughening kinetics of hydrogenated graphene**

S. Son^1, 2^, J. Figueira Nunes^3^, Y. Shin^3^, J-H. Lee^2, 4^ and C. Casiraghi* ^3^

^1^ School of Physics and Astronomy, University of Manchester, Manchester, M13 9PL, UK

^2^ National Graphene Institute, University of Manchester, Manchester, M13 9PL, UK

^3^ School of Chemistry, University of Manchester, Manchester, M13 9PL, UK

^4^ Department of Energy Systems Research and Department of Materials Science and Engineering, Ajou University, Suwon 16499, Republic of Korea

^*^corresponding author email: [cinzia.casiraghi@manchester.ac.uk](mailto:cinzia.casiraghi@manchester.ac.uk)

**Roughness profiles before hydrogenation**

Micro-mechanical exfoliation method was used to prepare the graphene and h-BN flakes. They are all produced in the same way and deposited on the same type of silicon substrate with 290 nm SiO_2_ (Gr/SiO_2_). In the case of the graphene/h-BN heterostructures (Gr/h-BN/SiO_2_), we selected 30-50 nm thick h-BN flakes in order to avoid any influence from the silicon roughness under the h-BN.

We characterized the individual RMS roughness by the AFM measurement before the hydrogenation process. The measurements are shown in Figure S1. The RMS roughness of h-BN/SiO_2_ and SiO_2_ area are ~0.07 nm and ~0.12 nm, respectively. The RMS roughness of Gr/h-BN/SiO_2_ and Gr/SiO_2_ area are ~0.1 nm and ~0.17 nm, respectively. The RMS-roughness results indicate that the h-BN flakes can provide a smoother substrate compared to silicon, in agreement with the literature.


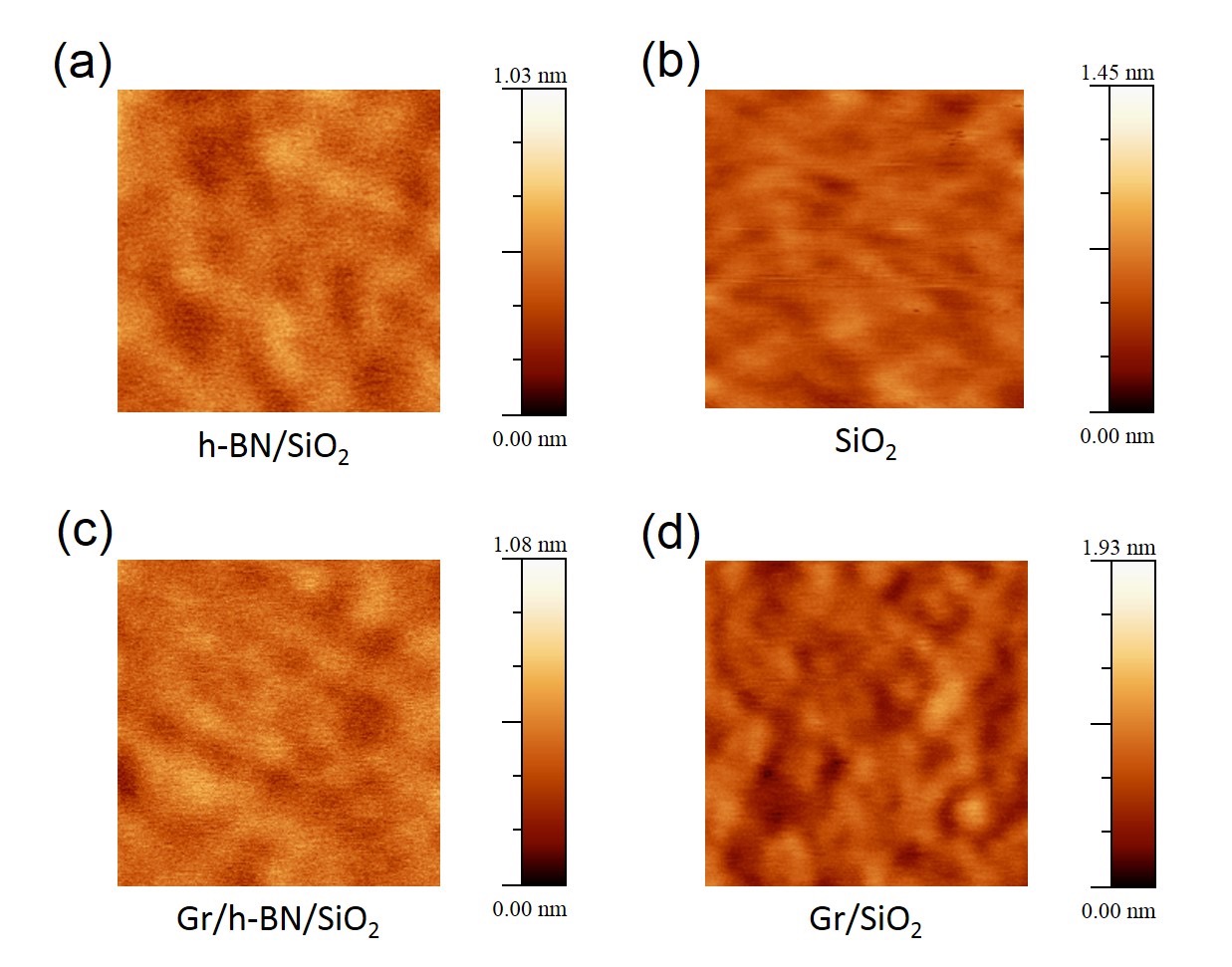


Figure S1. AFM images of different samples for the RMS roughness; (a) h-BN on the SiO_2_ substrate (RMS ~0.07 nm), (b) SiO_2_ substrate (RMS ~0.1 nm), (c) Gr/h-BN on the SiO_2_ substrate (RMS ~0.12 nm), and (d) Gr on the SiO_2_ substrate (RMS ~0.17 nm).
